# 5′-tRNA^HisGUG^ fragment: A preferred endogenous TLR7 ligand with reverse sequence activation insights

**DOI:** 10.1016/j.bpj.2025.04.027

**Published:** 2025-05-05

**Authors:** Kiran Bharat Lokhande, Ashutosh Singh, Rajan Vyas, Shreya Joe, Shailendra Asthana, Kamlesh Pawar

**Affiliations:** 1 Department of Life Sciences, School of Natural Science, Shiv Nadar Institution of Eminence Deemed to Be University, Greater Noida, India; 2 Centre of Excellence in Epigenetics, Department of Life Sciences, School of Natural Science, Shiv Nadar Institution of Eminence Deemed to Be University, Greater Noida, India; 3 Computational Biophysics and CADD Group, Computational and Mathematical Biology Center, Translational Health Science and Technology Institute, Faridabad, India

## Abstract

Toll-like receptor 7 (TLR7), a key member of the TLR family, plays a pivotal role in innate immunity, making it an attractive therapeutic target. However, current synthetic TLR7 agonists are often associated with significant toxicity, highlighting the need for safer, naturally occurring alternatives. Our recent research identified 5′-fragments of tRNA^HisGUG^ (5′-HisGUG) and tRNA^ValCAC/AAC^ (5′-ValCAC/AAC) as potent, naturally occurring TLR7 activators. While endogenous RNAs such as 5′-HisGUG are known to activate TLR7, the molecular details of their interaction remain unclear. To address this, we performed molecular dynamics simulations and MM/GBSA binding free energy analysis to investigate how these RNA fragments engage with TLR7 in comparison with synthetic agonists. Our results revealed that 5′-HisGUG, 5′-ValCAC/AAC, and reverse sequence (5′-HisGUG-Rev) exhibit strong binding affinities, with higher energetic favorability than synthetic agonists. The free energy fluctuations suggested that endogenous RNA ligands display greater conformational variability, possibly contributing to their activation potential. Notably, 5′-HisGUG-Rev effectively activated TLR7 and enhanced cytokine mRNA expression. Comparative analysis suggests that binding affinity alone does not directly predict activation, emphasizing the importance of both strong interaction and conformational flexibility in TLR7 activation. These findings position 5′-HisGUG as a promising natural TLR7 activator with potential therapeutic applications.

**Video Abstract:**

## Graphical abstract

**Figure:**
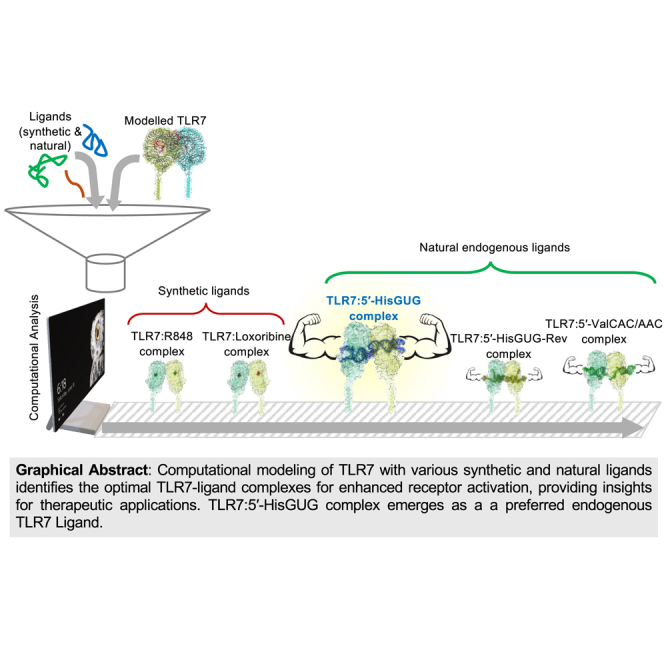

## Significance

Toll-like receptor 7 (TLR7) plays a vital role in immune defense but poses challenges for safe therapeutic targeting due to the toxicity of existing synthetic drugs. Through comparative computational analysis, we identified naturally occurring RNA fragments derived from tRNAs as promising candidates for effective and safe TLR7 activation. Specifically, fragments from tRNA^HisGUG^ and tRNA^ValCAC/AAC^ demonstrated strong binding and stabilization of TLR7, outperforming some synthetic activators. Advanced simulations and laboratory validation highlighted one fragment, 5′-HisGUG, as the most potent. This fragment significantly enhances immune responses, positioning it as a promising lead for safer therapeutic development.

## Introduction

The immune system recognizes both pathogen-associated molecular patterns (1,2) and damage-associated molecular patterns (3,4,5) via Toll-like receptors (TLRs) and other receptors to initiate protective responses (6,7). Surface TLRs such as TLR1, -2, -4, -5, -6, and -10 recognize bacterial, fungal, and viral components, while endosomal TLRs such as TLR3, -7, -8, and -9 detect nucleic acids (8). For instance, TLR7 and -8 detect single-stranded RNAs (ssRNAs), while TLR3 and -9 sense double-stranded RNAs and single-stranded DNAs, respectively. Activation of TLR7 or -8 leads to MyD88-dependent signaling, resulting in interferon and cytokine production (6,8). Two types of ssRNAs have been identified as ligands for TLR7 and TLR8: foreign ssRNAs originating from bacteria or viruses (9,10,11,12,13,14), and endogenous ligands such as miRNAs (15,16,17,18) and tRNA fragments (19,20) originating from host cells. The miRNA-mediated TLR7/8 activation is relevant not only to immune response (21,22) but also to neurodegeneration (15), cancers (23,24), autoimmunity (25,26), and various other diseases (27).

Recently, we showed that the 5′-fragments of tRNA^HisGUG^ (5′-HisGUG) and tRNA^ValCAC/AAC^ (5′-ValCAC/AAC), which are potent activators of TLR7, are present in extracellular vesicles (EVs) released by human macrophages. Their levels are significantly upregulated during mycobacterial infection, and they are found in much higher abundance than miRNAs (19,20). We observed that their levels are approximately 1000 times higher in the blood of infected patients compared with healthy individuals (20). This suggests that these endogenous TLR7 ligands are produced within host cells and play a crucial role in immune regulation. Although the precise ssRNA sequence determinants for TLR7 stimulation remain incompletely defined, it is established that successive U sequences and/or GU-rich sequences are essential for TLR7 activation. Structural analysis of TLR7 has revealed that G nucleotides (preferably G-cP) and ssRNAs containing successive Us serve as ligands for the first and second binding pockets of TLR7, respectively (10,28). Using mutation study, recently we reported that 5′-terminal GUUU sequencing of tRNA fragment is responsible for the activation of TLR7 (20). Although TLR7 is known to recognize the ssRNA as a ligand, a few synthetic molecules have been identified or as a ligand for TLR7. Imidazoquinoline derivatives and their counterparts serve as synthetic stimulants for TLR7/8, with one such derivative having gained FDA approval for treating topical antiviral conditions and skin cancer (29,30). They have been investigated for their potential as immunomodulatory agents against tumors and are being explored as potential vaccine adjuvants for both cancer and infectious diseases (29,31). Yet, the poor pharmacokinetic properties of imidazoquinoline derivatives and the toxicities linked to systemic usage pose significant challenges to their effective clinical adoption (29). Therefore, it is important to identify endogenous ligands for TLR7 which could potentially have less toxicity compared with the synthetic compounds.

TLR7 is involved in various biological processes and diseases (32,33,34,35,36,37). For example, *TLR7* mutations are associated with the symptom severity of COVID-19 (38,39,40). TLR7 has also been implicated in the progression of Parkinson’s disease (41), Alzheimer’s disease (15,42,43), and autoimmune diseases such as systemic lupus erythematosus (44) and atherosclerosis (45,46). Hence, it is crucial to comprehend both the ligands of TLRs and their mechanisms of interaction. As the mounting evidence implicating TLR7 in the pathogenesis of noninfectious diseases emphasizes the need for a more profound understanding of endogenous ssRNA ligands of immune receptors, the exploration and characterization of these ligands are still in their early stages. We are only beginning to recognize previously concealed classes of sncRNAs that were undetectable via standard RNA-seq methods.

In silico approaches offer a robust platform to elucidate the detailed interactions between protein and its ligands, which can be laborious and challenging to decipher experimentally. By leveraging high-resolution structural data from the Protein Data Bank (PDB), computational modeling and molecular docking techniques allow for detailed analysis of protein in complex with various ligands. For instance, in this study, we utilized the PDB structure 7CYN to model the complete dimer form of TLR7 (47), addressing missing loop regions to ensure structural integrity. Docking studies with known ligands such as PolyU RNA (48), loxoribine (49), and R848 (50) were performed using the Glide program to predict their binding modes within the TLR7 binding cavity. Additionally, RNA (5′-fragment of tRNA^HisGUG^ and tRNA^ValCAC^ and other small noncoding RNAs) docking provided insights into the interactions between TLR7 and ssRNAs, enabling us to identify key binding residues and understand binding affinities. These computational techniques, coupled with full atomistic molecular dynamics (MD) simulations, allowed us to monitor the dynamic behavior of TLR7-ligand complexes over extended periods. This combined approach not only enhances our understanding of TLR7-5′-tRNA fragments interactions at the atomic level but also aids in identifying novel endogenous ligands with potential therapeutic applications, facilitating new pathways in immunomodulatory treatments. This knowledge aids in the identification of potential unidentified ssRNAs through screening and comparison with current signature patterns.

## Materials and methods

### Retrieval of TLR7 dimer structure and its *in silico* preparation

The structure of TLR7 in its dimer form is available in the PDB database (https://www.rcsb.org/). We retrieved this cryo-EM dimer from the PDB database with the PDB: 7CYN (47), which has a resolution of 4.20 Å. We selected this TLR7 dimer because it includes the transmembrane region. After retrieval, we analyzed this structure using the Maestro software (51) and observed that it contains some missing loop information. Specifically, two missing loops were identified in both chains (chain A and chain B) of this PDB: 7CYN. The missing loops span the regions Pro435 to Lys470 and Cys475 to Ser490 of TLR7.

### TLR7 loop modeling

The missing loops were modeled using the SWISS-MODEL Server (https://swissmodel.expasy.org/). For this, the amino acid sequence of TLR7 was obtained from the UniProt database (https://www.uniprot.org/) with UniProt ID Q9NYK1. We used the User Template mode of SWISS-MODEL to ensure the original protein structure was maintained while modeling only the missing loops. The PDB structure 7CYN was provided as the template. This approach allowed SWISS-MODEL to preserve the intact portions of the protein and focus solely on the missing parts of the structure. The resulting modeled structures were then subjected to protein preparation using Maestro software to ensure structural correctness. This involved optimizing hydrogen bonds and minimizing the energy of heavy atoms using the OPLS-2005 force field. This process resulted in high-confidence structures suitable for further studies.

### RNA structure prediction and retrieval of active ligands

In this study, we used ssRNA40-M as a negative control RNA and ssRNA40 as a positive control RNA. Additionally, we tested three RNAs (5′-ValCAC/AAC, 5′-HisGUG, and 5′-HisGUG-Rev). 5′-HisGUG-Rev has a reversed RNA sequence of 5′-HisGUG. The sequences are provided in Table 1. For 3D RNA structure prediction, we used the 3dRNA webserver (http://biophy.hust.edu.cn/new/3dRNA/create). The 3dRNA server generates 1000 candidate structures for a given target RNA. These candidates are then classified into clusters using the DBSCAN clustering method. The five largest clusters are selected and scored by 3dRNAscore, which is an all-atom statistical potential scoring function that evaluates atom-atom distances and backbone dihedral angles. From each cluster, the model with the best score is chosen, resulting in five final models (52). The structures of TLR7 in complex with its ligands have been reported. These ligands, known to activate TLR7, can serve as positive controls for predicting RNA-protein interactions. The ligands include PolyU RNA, loxoribine, and R848. We retrieved loxoribine (PubChem CID: 135410906) and R848 (PubChem CID: 159603) from the PubChem database (https://pubchem.ncbi.nlm.nih.gov/). PolyU RNA was retrieved from the PDB database, where it is available in complex with monkey (*Macaca mulatta*) TLR7 (PDB: 5GMF) (10). All small molecules were then subjected to energy minimization using the OPLS-2005 force field in Maestro software.

**Table 1:** Sequences of RNA used for RNA structure prediction

RNA	Sequence
ssRNA40-M	GCCCGACAGAAGAGAGACAC
ssRNA40	GCCCGUCUGUUGUGUGACUC
5′-ValCAC/AAC	GUUUCCGUAGUGUAGUGGUUAUC ACGUUCGCCU
5′-HisGUG	GCCGUGAUCGUAUAGUGGUUAGUA CUCUGCGUUG
5′-HisGUG-Rev	GUUGCGUCUCAUGAUUGGUGAUAU GCUAGUGCCG

### Binding site definition

For defining the binding site, we used information from the TLR7-PolyU complex available in the PDB database with the ID 5GMF, as mentioned above. The PDB structure used in this study, PDB: 7CYN, does not contain any ligand bound to it. To identify the binding site residues in 7CYN, we superimposed the structure of TLR7 from 5GMFonto 7CYN. This superimposition was possible because both structures are very similar and present in dimeric form. The key difference between the two is that 5GMFdoes not contain the transmembrane region but includes the PolyU small molecule, while 7CYN contains the transmembrane region but no small molecules. By superimposing these structures, we were able to transfer the PolyU binding site information to 7CYN, thus obtaining precise binding site information for the 7CYN structure. From this exercise, we identified the following residues to define the binding site for TLR7, which will be used for both small molecule and RNA docking. For chain A: Arg97, Glu156, Gln181, Tyr184, Arg186, Arg467, Asp469, Arg473, and Cys575. For chain B: Arg97, Glu156, Gln181, Tyr184, Arg186, Arg467, Asp469, Arg473, Cys575, Lys688, and Arg636.

### Small-molecule docking using Glide

For docking the small molecule PolyU RNA, loxoribine, and R848, we used the Glide program of Schrödinger software to predict their binding modes within the TLR7 binding cavity. The binding site was defined using the residues identified above, and we set up the grid box for docking using the Glide grid program (53). After preparing the grid box, we first performed standard precision docking to obtain the initial binding poses of the ligands within the cavity. We then subjected these initial poses to extra precision docking to achieve more accurate binding poses (54). Finally, we selected the best poses from the extra precision docking for further calculations.

### RNA docking using HDOCK

For the protein-RNA docking of TLR7 with the RNA structures, we used the HDOCK online server (http://hdock.phys.hust.edu.cn/). HDOCK predicts binding complexes and binding affinities by modeling intermolecular interactions at the interface of two proteins or protein-RNA or protein-DNA complexes using a hybrid algorithm (55). The algorithm performs rigid docking, treating both the receptor and the ligand as rigid molecules during the docking process. We conducted macromolecular docking of TLR7 with different RNA structures, initially focusing on chain A. Subsequently, we performed macromolecular docking for chain B of TLR7 to obtain RNA binding poses in the dimeric form of TLR7. We obtained all docked complexes from the HDOCK server and analyzed their binding modes using Maestro. We performed molecular docking twice for both the Glide and HDOCK programs. As we are analyzing the dimer structure of TLR7, we first docked the ligands and RNA within the binding pocket of TLR7 chain A using both programs. Next, we repeated the docking process for TLR7 chain B using the same protocol. This dual docking approach was conducted to obtain binding poses of the ligands within the dimeric structure of TLR7. Finally, we selected the poses of the ligands and RNA that docked in a similar manner in both binding pockets. We focused on the orientation of the ligands and RNA in the binding pockets and selected the poses that were consistent across both pockets of TLR7 for further analysis. This docking serves only as an initial approximation, and MD simulations refine the binding poses by incorporating RNA flexibility.

### Full atomistic MD simulation

To assess the binding strength of the small molecules PolyU RNA, loxoribine, and R848, as well as the RNAs (ssRNA40, ssRNA40-M, 5′-ValCAC/AAC, 5′-HisGUG, and 5′-HisGUG-Rev) with TLR7, and to reveal the dynamic behavior of TLR7, we conducted 500 ns of full atomistic MD simulations using Desmond software (56). We conducted a total of nine simulations, which included the complexes of TLR7 with small molecules and RNAs, as well as the simulation of TLR7 in its apo form. The docked complexes were prepared for MD simulation using the system builder panel in Desmond software. Each complex was immersed in a solvated orthorhombic box measuring 10 × 10 × 60 Å, using the TIP3P water solvent model. Since TLR7 is a transmembrane protein, dipalmitoylphosphatidylcholine bilayers were introduced at the specified transmembrane regions for both chain A and chain B of TLR7. Counter ions (Na^+^/Cl^−^) were added to the solvated system to neutralize the overall charge of each complex. The first 2 ns of the simulation were used for equilibration. During this phase, the system was gradually relaxed, allowing temperature, pressure, and density to stabilize. This phase is not included in the final statistical analysis. The subsequent 500 ns was considered the production phase. For the 500 ns MD simulation, we employed the steepest descent energy minimization method using the OPLS-2005 force field. All systems were simulated using the NPT ensemble, maintaining a temperature of 300 K and a pressure of 1 bar. The Nose-Hoover chain thermostat method was used to control the temperature, and the Martyna-Tobias-Klein barostat method was used to regulate the pressure throughout the 500 ns simulation. Short-range interactions, specifically Coulombic interactions, were calculated within a cutoff radius of 9.0 Å. The equations of motion were integrated using the leap-frog algorithm with a time step of 2 fs. Throughout the simulation, we monitored structural changes in the complex system by comparing them with the conformation of the docked complex and the TLR7 apo form. The binding free energy (ΔG) of the TLR7-ligand complexes was calculated using the MM/GBSA method in Schrödinger’s Prime module based on snapshots extracted from the 500 ns MD trajectory. The average ΔG and standard deviation were computed to assess ligand stability and binding strength, providing a more accurate measure of ligand affinity beyond docking scores.

### In vitro RNA synthesis

In vitro transcription was employed to prepare synthetic 5′-tRNA halves according to a previously described method (57). The dsDNA templates were synthesized using T4 DNA polymerase (New England Biolabs, UK) and overlapping forward and reverse primers. Native PAGE was performed using 40% 19:1 acrylamide, *N*,*N*,*N*′,*N*′-tetramethylethylenediamine (TEMED) and ammonium persulfate (APS) to confirm the success of the reaction. The templates were subjected to in vitro transcription using T7 RNA polymerase (gifted by Prof. Sanjeev Galande) in a reaction carried out at 37°C for 10 h. The synthetic RNAs were then resolved using a denaturing PAGE with single-nucleotide resolution. This was prepared using 12% 7 M urea solution, TEMED, and APS, and run using 1X Tris-borate-EDTA buffer at 200 V. The gel was stained with SYBRGold visualizing dye (Thermo Fisher Scientific, USA). Gel-extracted RNA was purified using a gel extraction buffer and filtered. After purifying RNA and subjecting it to urea PAGE separation, a distinct RNA band was visualized through ethidium bromide (Bio-Rad, USA) staining.

### Transfection of RNA

THP-1 human acute monocytic leukemia cells (American Type Culture Collection, USA) were cultured in RPMI 1640 medium (Corning, USA) with 10% FBS and differentiated into human monocyte-derived macrophages (HMDMs) using phorbol 12-myristate 13-acetate (Sigma-Aldrich, USA), as described previously (19,58). Before transfection, the cells were primed with 100 units/mL of interferon-γ (Thermo Fisher Scientific, USA) for 18–24 h (59). To deliver RNAs to endosomes, we used the cationic liposome 1,2-dioleoyloxy-3-trimethylammonium-propane (DOTAP) (Sigma-Aldrich, USA) as previously described (19,60,61). In brief, 230 pmol of synthetic RNAs was mixed with 60 *μ*L of HBS buffer and 15 *μ*L of DOTAP reagent and incubated for 15 min. The RNA-DOTAP solution was then added to 1 mL RPMI 1640 medium with 2% FBS, followed by incubation of the cells for 6 and 12 h. Expression of cytokine mRNAs were measured using standard RT-qPCR.

### Quantification of mRNAs by standard RT-qPCR

Total RNA was isolated from the cells using TRIsure (Bioline, UK). For quantification of mRNAs by standard RT-qPCR, total RNA was treated with DNase I (Promega, USA) and subjected to reverse transcription using RevertAid Reverse Transcriptase (Thermo Fisher Scientific, USA) and a reverse primer. The synthesized cDNAs were then subjected to PCR using 2×qPCR Master Mix (Bioland Scientific, USA) and forward and reverse primers (19).

## Results

### Modeling the missing loops resulted in a complete dimer structure of TLR7

**Figure 1:**
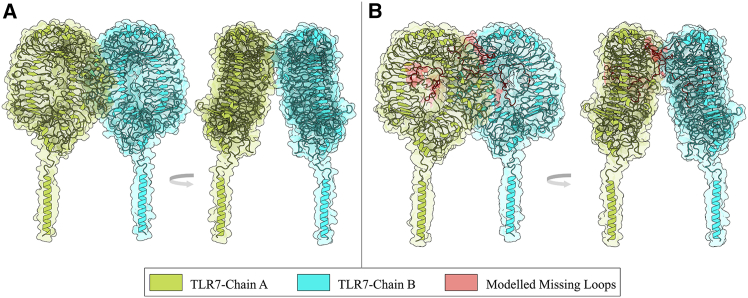
The dimer structure of TLR7. (*A*) The cryo-EM dimer structure of TLR7 obtained from the PDB database and (*B*) The loop-modeled structure of TLR7 using the SWISS-Model server.

The TLR7 structure utilized in this study (PDB: 7CYN) has two missing loop regions. These loops span the regions from Pro435 to Lys470 and Cys475 to Ser490 of TLR7, encompassing a total of 51 missing residues. These missing loops were modeled using the SWISS-MODEL server, and the modeled structure was used for further study. Fig. 1 shows both the available structure (Fig. 1 *A*) and the loop modeled structure of TLR7 (Fig. 1 *B*). The missing residues form a disordered loop region in TLR7, which plays a crucial role in ligand or RNA binding. Importantly, one of these loops is located at the interface of the TLR7-TLR7 dimer, as evident from Fig. 1.

### 3dRNA generated 3D RNA structures and comparative stability scores across different RNA molecules

We used the 3dRNA webserver to predict the 3D structures of the control and 5′-tRNA fragments. The server generated 1000 candidate structures for each RNA, which were then classified into clusters using the DBSCAN clustering method. The five largest clusters were selected and scored by 3dRNAscore, an all-atom statistical potential scoring function that evaluates atom-atom distances and backbone dihedral angles. The best-scoring model from each cluster was chosen, resulting in five final models per RNA (Table 2).

**Table 2:** The detailed 3dRNAscores for each RNA

RNA	Scores by 3dRNAscore (the lower the better)				
	No. of model (1–5) ranking from highest to lowest				
ssRNA40-M	28.4	29.3	29.3	29.3	30.1
ssRNA40	26.7	28.5	29.1	30.9	33.4
5′-ValCAC/AAC	26.7	26.9	27.1	27.3	27.7
5′-HisGUG	27.2	27.4	27.4	27.6	27.6
5′-HisGUG-Rev	28.0	28.2	29.3	30.0	31.0

**Figure 2:**
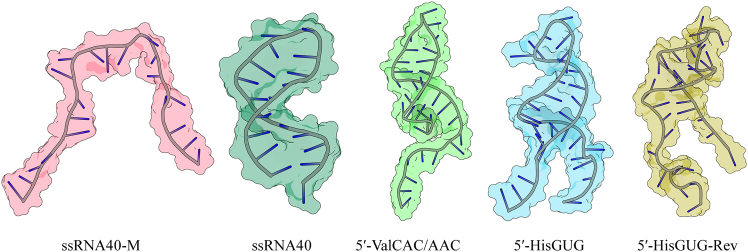
Best 3dRNA predicted models for ssRNA40-M, ssRNA40, 5′-ValCAC/AAC, 5′-HisGUG, and 5′-HisGUG-Rev. All RNAs formed secondary structures, except for ssRNA40-M, a negative control RNA, which remained single-stranded throughout its entire sequence.

The ssRNA40-M predictions yielded scores between 28.4 and 30.1, with the optimal model scoring 28.4. For ssRNA40, scores ranged from 26.7 to 33.4, with the best score at 26.7, suggesting a potentially more favorable conformation than ssRNA40-M. The 5′-ValCAC/AAC models had scores between 26.7 and 27.7, with the lowest score at 26.7, indicating high structural stability, comparable with ssRNA40. 5′-HisGUG showed a tighter score range (27.2–27.6), with a minimum score of 27.2, implying consistent stability. In contrast, 5′-HisGUG-Rev had a broader range (28.0–31.0), with the best score at 28.0, indicating moderate stability. Overall, 5′-ValCAC/AAC has the most stable predicted structure among the 5′-tRNA fragments, followed closely by 5′-HisGUG, while 5′-HisGUG-Rev showed the least stability. For further analysis, we selected the best model for each RNA, as shown in Fig. 2, based on the lowest 3dRNAscore, for subsequent docking and MD simulations with TLR7, which will inform experimental validation and docking studies with TLR7.

### The superimposition of human and monkey TLR7 structures revealed the binding site information in human TLR7

**Figure 3:**
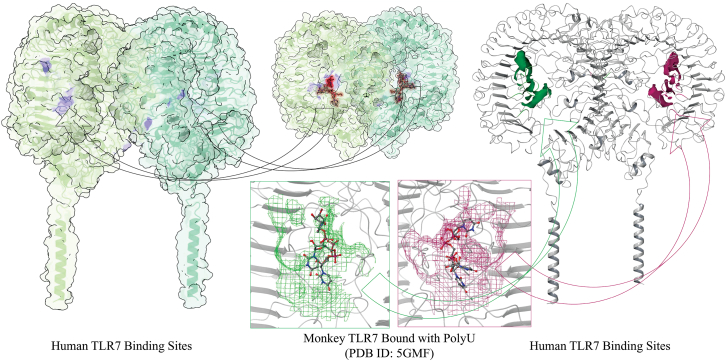
Transformation of binding site information from monkey TLR7 to human TLR7. The bound PolyU with monkey TLR7 is represented in a ball-and-stick model in red, shown in both classic cartoon form and molecular surface view in the middle panel. The transformed binding site from monkey TLR7 to human TLR7 is depicted in the left and right panels. In the left panel, the binding site residues are shown in purple in a molecular view with a molecular surface representation. In the right panel, the binding site residues are displayed in green for chain A and pink for chain B, shown in classic cartoon form.

We aimed to transfer the binding site information from monkey TLR7 to human TLR7. The PolyU ligand bound to monkey TLR7 is represented in red as a ball-and-stick model in both classic cartoon form and molecular surface view in the middle panel of Fig. 3. The binding site residues identified in monkey TLR7 were transformed and applied to human TLR7. This transformation is illustrated in the left and right panels of Fig. 3. To validate the transformation of the binding site, we performed a protein structural alignment between monkey TLR7 and human TLR7. The alignment score obtained was 0.33 (with smaller values indicating better alignment), and the root mean-square deviation (RMSD) was 2.41 Å. The slightly higher RMSD value is attributed to the presence of the transmembrane region in human TLR7, which is absent in monkey TLR7.

### Molecular docking of TLR7 with various ligands provided insights into the comparative binding patterns across the different complexes

The Glide docking results for TLR7 with small molecules (PolyU RNA, loxoribine, and R848) demonstrate distinct binding affinities and interaction profiles within the binding pockets of chains A and B of the TLR7 dimer (Table 3). Among these, PolyU RNA exhibited the strongest binding affinity, with docking scores of −8.4 kcal/mol (chain A) and −7.5 kcal/mol (chain B). Loxoribine followed with moderate binding affinity (−5.8 and −4.7 kcal/mol, respectively), while R848 showed the weakest affinity (−3.9 kcal/mol for both chains). The superior binding of PolyU RNA suggests stronger stabilization within the TLR7 binding site, whereas the weaker docking score of R848 indicates less favorable interactions with the receptor.

**Table 3:** Detailed intermolecular interactions between TLR7 and small molecules

Complex	TLR7-chain A	Type of interaction	TLR7-chain B	Type of interactions
7CYN-PolyU	Asn479	HBond	Asn479	HBond
	Lys212	HBond	Lys212	HBond
	Lys470	HBond	Arg473	HBond
	Tyr233	HBond	Lys470	HBond
	Asp469	HBond	Tyr233	HBond
	Asn234	HBond	Lys470	salt bridge
	Arg467	HBond	Asp469	HBond
	Arg296	salt bridge		
	Glu318	HBond		
7CYN-Loxoribine	Tyr233	HBond	Tyr233	HBond
	Asn479	HBond	Asn479	HBond
	Glu481	2 HBond	Glu481	HBond
	Lys470	Pi-cation	Lys470	Pi-cation
	Ala472	HBond	Ala472	HBond
	Asp469	HBond	Asp469	2 HBond
7CYN-R848	Arg467	HBond	Lys212	2 Pi-cation
	Asp469	HBond	Asp469	HBond
	Ala472	HBond	Ala472	HBond
			Glu481	HBond

All intermolecular interactions (H-bonds, salt bridges, π-cation, and π-π stacking) were identified using Schrödinger Maestro’s interaction analysis tool. The intermolecular interaction analysis (Table 3) reveals that PolyU RNA establishes multiple hydrogen bonds (H-bonds) with key residues such as Asn479, Lys212, and Asp469 in both chains, in addition to salt bridges (Arg296, Lys470), which contribute to its stable binding. Loxoribine interacts via H-bonds with Tyr233, Asn479, and Glu481, and π-cation interactions with Lys470, highlighting the role of electrostatic interactions in stabilizing its binding. In contrast, R848, which has the weakest binding affinity, forms fewer H-bonds (Arg467, Asp469, Ala472) and π-cation interactions with Lys212, suggesting that reduced stabilization may account for its lower docking score.

**Figure 4:**
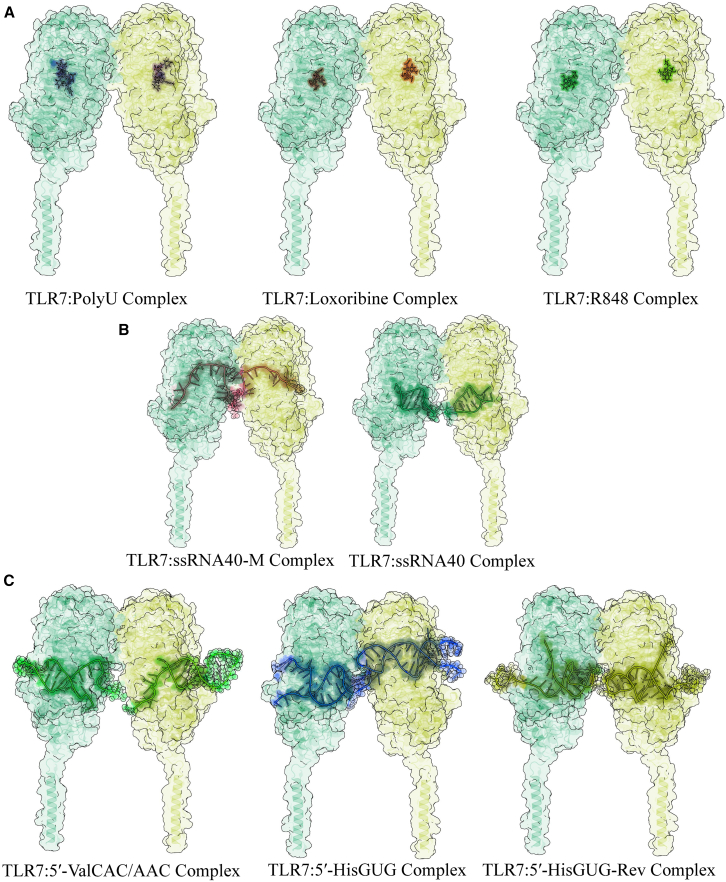
Illustration of the docked poses of small molecules or ssRNA with the TLR7 dimer. In each panel, the TLR7 dimer is composed of chain A, shown as a molecular surface in ineffable green, and chain B in olive yellow. The small molecules are represented as ball-and-stick models and the RNAs are represented as tube models. (*A*) PolyU is located within the cavity of TLR7, interacting with both chain A and chain B. Similarly, loxoribine is positioned inside the TLR7 cavity, engaging both chains. R848 occupies the same cavity within the TLR7 dimer, also interacting with both chain A and chain B. (*B*) ssRNA40-M is embedded within the cavity of TLR7, interacting with both chain A and chain B. Likewise, ssRNA40 is similarly positioned within the TLR7 cavity, engaging both chains. (*C*) 5′-ValCAC/AAC is nestled within the cavity of TLR7, interacting with both chain A and chain B. Similarly, 5′-HisGUG is positioned inside the TLR7 cavity, engaging both chains. 5′-HisGUG-Rev occupies the same cavity within the TLR7 dimer, also interacting with both chain A and chain B.

Fig. 4*A* demonstrates the binding poses of the PolyU, loxoribine, and R848 with the TLR7 dimer. PolyU is observed to be buried within the cavity of the TLR7 dimer, interacting with both chain A (*ineffable green*) and chain B (*olive yellow*). Similarly, loxoribine and R848 are positioned inside the TLR7 cavity, engaging both chains, indicating the significant binding interactions of these molecules with the receptor. Fig. 4 *B* demonstrates the binding pose of ssRNA40-M and ssRNA40 with the TLR7 dimer. The ssRNA40-M is observed to be buried within the cavity of the TLR7 dimer, interacting with both chain A (*ineffable green*) and chain B (*olive yellow*). Similarly, ssRNA40 is positioned inside the TLR7 cavity, engaging both chains, indicating the significant binding interactions of these RNAs with the receptor. Fig. 4 *C* demonstrates the interaction binding poses of 5′-ValCAC/AAC, 5′-HisGUG, and 5′-HisGUG-Rev with the TLR7 dimer. 5′-ValCAC/AAC is observed to be buried within the cavity of the TLR7 dimer, interacting with both chain A (*ineffable green*) and chain B (*olive yellow*). Similarly, 5′-HisGUG and 5′-HisGUG-Rev are positioned inside the TLR7 cavity, engaging both chains, indicating the significant binding interactions of these RNAs with the receptor. Figs. 1, 2, 3, and 4 are generated from Protein Imager Server (https://3dproteinimaging.com/protein-imager/).

Similarly, docking analysis of TLR7 with RNA ligands (ssRNA40-M, ssRNA40, and 5′-tRNA fragments) revealed significant variations in binding affinity and interaction strength (Table 4). The ssRNA40-M complex displayed the strongest binding affinity, particularly in chain A (−253.1 kcal/mol) compared with chain B (−138.7 kcal/mol), followed by ssRNA40 (−163.1 and −78.5 kcal/mol, respectively). The 5′-ValCAC/AAC complex exhibited preferential binding in chain B (−208.6 kcal/mol), while 5′-HisGUG and 5′-HisGUG-Rev showed the weakest binding, with less-favorable docking scores (Table S1). Interaction analysis (Table 4) suggests that ssRNA40-M forms extensive stabilizing interactions, including multiple H-bonds with Thr39, Lys470, and Lys502, and salt bridges with Lys502, His630, and Lys659, all contributing to its superior binding. The ssRNA40 complex similarly interacts via H-bonds with Arg473, Met485, and Lys659, in addition to salt bridges involving Lys731, which provide further stabilization. In contrast, 5′-HisGUG and 5′-HisGUG-Rev rely more on π-π stacking interactions (Tyr468, Phe349) rather than H-bonds, correlating with their weaker docking scores. These results indicate that binding affinity is not solely determined by docking scores but also by the number and nature of stabilizing intermolecular interactions, particularly hydrogen bonding, salt bridges, and π-cation interactions.

**Table 4:** Detailed intermolecular interactions between TLR7 and RNAs

Complex	TLR7-chain a	Type of interactions	TLR7-chain B	Type of interactions
7CYN-ssRNA40-M	Thr39	HBond	Tyr233	2 HBond
	Lys470	HBond	Arg553	HBond
	Lys502	HBond	His630	salt bridge
	Lys502	salt bridge	Lys108	HBond
	Lys659	HBond	Lys212	HBond
	Lys776	HBond	Lys212	2 Pi-cation
	Val38	2 HBond	Glu453	HBond
	Lys684	Pi-cation	Ser454	HBond
	Arg473	Pi-cation	Tyr465	2 Pi-Pi stacking
	Arg376	Pi-cation	Lys659	2 Pi-cation
	Phe484	Pi-Pi stacking		
7CYN-ssRNA40	Arg473	salt bridge	Thr71	HBond
	Met485	HBond	Arg97	HBond
	Lys600	salt bridge	Tyr471	HBond
	Lys659	salt bridge	Arg473	HBond
	Lys659	HBond	Lys684	HBond
	Lys684	Pi-cation	Lys659	HBond
	Gln710	HBond	Lys659	salt bridge
	Lys688	salt bridge		
	Lys731	2 salt bridge		
	Lys731	HBond		
7CYN-5′-ValCAC/AAC	Asp37	HBond	Asn488	HBond
	Thr52	HBond	Tyr4638	HBond
	Arg97	salt bridge	Asn479	HBond
	Arg473	salt bridge	Arg473	salt bridge
	Lys478	HBond	His708	HBond
	Lys44	2 salt bridge	Lys684	2 Pi-cation
	Thr810	2 HBond	Ala683	HBond
	His782	Pi-Pi stacking	Lys731	salt bridge
	Ser756	HBond	Asp705	HBond
	Lys731	salt bridge		
	Lys684	HBond		
7CYN-5′-HisGUG	Leu40	HBond	Arg467	HBond
	Thr52	HBond	Tyr468	3 Pi-Pi stacking
	His782	HBond	Tyr468	HBond
	Ser756	HBond	Arg473	2 salt bridge
	Lys731	2 salt bridge	Gln458	HBond
	Lys731	HBond	His464	HBond
	Arg473	2 salt bridge	Lys424	Pi-cation
	Meth485	HBond	Arg376	salt bridge
	Glu453	HBond	Phe349	Pi-Pi stacking
	Ser451	HBond	Lys480	Pi-cation
	Arg553	HBond		
	Arg553	salt bridge		
7CYN-5′-HisGUG-Rev	Lys54	HBond	Lys478	salt bridge
	Arg473	HBond	Lys731	salt bridge
	His708	HBond	Lys776	2 salt bridge
	Lys684	salt bridge	Lys776	HBond
	Lys659	salt bridge		
	Arg627	2 salt bridge		
	Glu453	HBond		
	Arg467	HBond		
	Arg467	Pi-cation		
	Arg296	HBond		
	Lys373	HBond		

### MD simulation: RMSD and RMSF analyses reveal ligand-specific variations for TLR7

**Figure 5:**
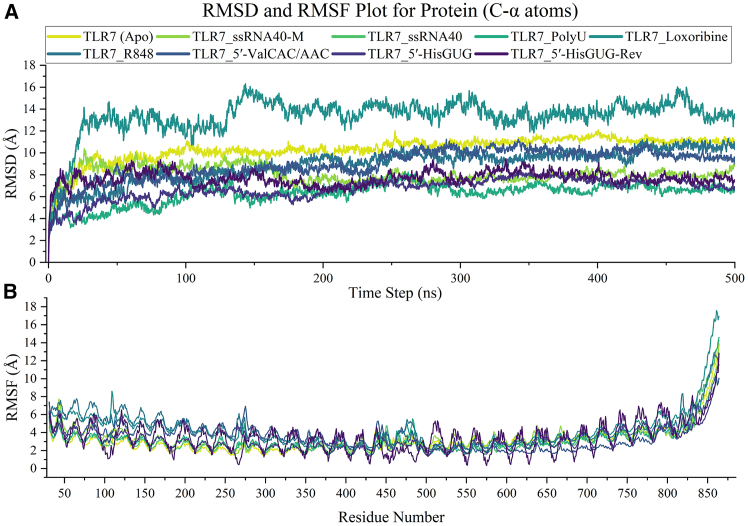
RMSD and RMSF plot of C-α atoms for TLR7 during 500 ns of MD simulations for different complexes. (*A*) The RMSD plot compares the structural stability of TLR7 in its apo form and when complexed with various small molecules (PolyU, loxoribine, R848) and RNAs (ssRNA40-M, ssRNA40, 5′-ValCAC/AAC, 5′-HisGUG, 5′-HisGUG-Rev). Lower RMSD values indicate greater stability, with notable stability observed for TLR7 complexes with PolyU RNA and ssRNA40. (*B*) The RMSF plot compares the structural flexibility of TLR7 in its apo form and when complexed with various small molecules (PolyU, loxoribine, R848) and RNAs (ssRNA40-M, ssRNA40, 5′-ValCAC/AAC, 5′-HisGUG, 5′-HisGUG-Rev). Lower RMSF values indicate greater stabilization, with notable stabilization observed for TLR7 complexes with PolyU RNA and ssRNA40.

The RMSD values of the C-α atoms for TLR7 were analyzed over 500 ns of MD simulations for various complexes, including small molecules and RNAs. Fig. 5 *A* shows the RMSD plot of TLR7 C-α atoms for different complexes during 500 ns of MD simulations. The average root mean-square deviation (RMSD) and root mean-square fluctuation (RMSF) values with standard deviations for each complex are provided in Tables S2 and S3.

The apo form of TLR7 exhibited the highest average RMSD (10.3 Å) with a standard deviation of 1.2 Å, indicating significant conformational deviations in the absence of any ligand or RNA. This high RMSD value suggests that TLR7 in its unbound state undergoes substantial structural fluctuations, implying increased flexibility compared with the ligand-bound states. The TLR7 complexed with ssRNA40-M showed an average RMSD of 8 Å and a lower standard deviation of 0.7 Å, suggesting reduced structural fluctuations compared with the apo form. The decrease in RMSD indicates that the presence of ssRNA40-M contributes to reduced TLR7 conformation, although not as effectively as ssRNA40 or PolyU RNA. The ssRNA40 complex exhibited an average RMSD of 6.2 Å with a standard deviation of 0.9 Å, comparable with the PolyU RNA complex (average RMSD of 6.2 Å, standard deviation of 0.9 Å), suggesting that these RNAs interact effectively with TLR7 and help maintain a more stable conformation during the simulation.

Among the small-molecule complexes, TLR7 bound to loxoribine had the highest average RMSD (13.3 Å) with a standard deviation of 1.7 Å, indicating significant structural fluctuations. This suggests that loxoribine binding does not restrict TLR7’s conformational variability as effectively as RNA ligands. The TLR7-R848 complex exhibited an average RMSD of 8.9 Å with a standard deviation of 1.4 Å, reflecting moderate conformational changes during the simulation. While R848 interacts with TLR7, its effect on conformational stabilization appears to be less pronounced than that of PolyU RNA or ssRNA40. The TLR7-5′-ValCAC/AAC complex showed an average RMSD of 8.9 Å with a standard deviation of 1.4 Å, suggesting a similar degree of structural fluctuations as TLR7-R848. In contrast, the TLR7-5′-HisGUG complex had an average RMSD of 6.7 Å with a standard deviation of 0.9 Å, which is comparable with the ssRNA40 and PolyU RNA complexes. Despite its higher docking score, the relatively lower RMSD value suggests strong interactions and a more restricted conformational state upon binding to TLR7.

For the TLR7-5′-HisGUG-Rev complex, the average RMSD was 7.6 Å with a standard deviation of 0.6 Å, indicating moderate structural fluctuations. This suggests that, while 5′-HisGUG-Rev interacts with TLR7, its conformational impact is intermediate between the most stable RNA complexes and other small-molecule-bound states.

The RMSF values of the C-α atoms for TLR7 were analyzed over 500 ns of MD simulation for various complexes, including small molecules and RNAs. Fig. 5 *B* shows the RMSF plot of TLR7 C-α atoms for different complexes during 500 ns of MD simulations. The apo form of TLR7 exhibited an average RMSF of 3 Å with a standard deviation of 1.4 Å, indicating baseline flexibility. This suggests that, in the absence of any ligand or RNA, TLR7 exhibits moderate fluctuation, which reflects the natural mobility of the protein regions. The complex with ssRNA40-M showed an average RMSF of 3.4 Å and a standard deviation of 1.3 Å. This indicates slightly higher flexibility compared with the apo form. The increased flexibility suggests that the ssRNA40-M binding does not significantly stabilize TLR7, allowing for moderate fluctuations. The ssRNA40 exhibited an average RMSF of 3 Å and a standard deviation of 1.6 Å, similar to the apo form. The comparable RMSF values indicate that the ssRNA40 maintains the natural mobility of TLR7 while providing sufficient stabilization. PolyU RNA demonstrated an average RMSF of 3.1 Å with a standard deviation of 1.5 Å. This indicates slightly higher flexibility than the ssRNA40 but still within a similar range, suggesting that PolyU RNA provides good stabilization while allowing necessary protein flexibility. The complex with loxoribine had the highest average RMSF of 4.2 Å and the largest standard deviation of 2.1 Å among all complexes, indicating significant flexibility and potential instability. The high RMSF value suggests that loxoribine binding does not effectively stabilize TLR7, leading to considerable fluctuations in the protein structure. The RMSF for TLR7-R848 complex was 4.1 Å with a standard deviation of 1.7 Å, suggesting higher flexibility compared with the apo form and other complexes. This indicates that R848 also does not stabilize TLR7 effectively, resulting in increased mobility of the protein regions. The TLR7-5′-ValCAC/AAC complex showed an average RMSF of 3.5 Å and a standard deviation of 1.4 Å, indicating moderate flexibility. The RMSF values suggest that 5′-ValCAC/AAC provides some stabilization to TLR7, but not as effectively as the ssRNA40 or PolyU RNA. 5′-HisGUG had an average RMSF of 3.1 Å and a standard deviation of 1.2 Å, suggesting relatively low flexibility and good stabilization, comparable with the ssRNA40 and PolyU RNA. Despite its higher docking score, the low RMSF value indicates strong interactions and effective stabilization of TLR7 by 5′-HisGUG. The average RMSF for 5′-HisGUG-Rev was 3.4 Å with a standard deviation of 1.6 Å, indicating reasonable flexibility, better than 5′-ValCAC/AAC and TLR7-R848 complexes but less stable than 5′-HisGUG. The RMSF values suggest that 5′-HisGUG-Rev interacts with TLR7 with moderate stabilization, allowing some necessary protein flexibility.

### The RMSD values obtained from the monomer units of TLR7 differ from those produced after the dimeric TLR7 interacts with its ligands

**Figure 6:**
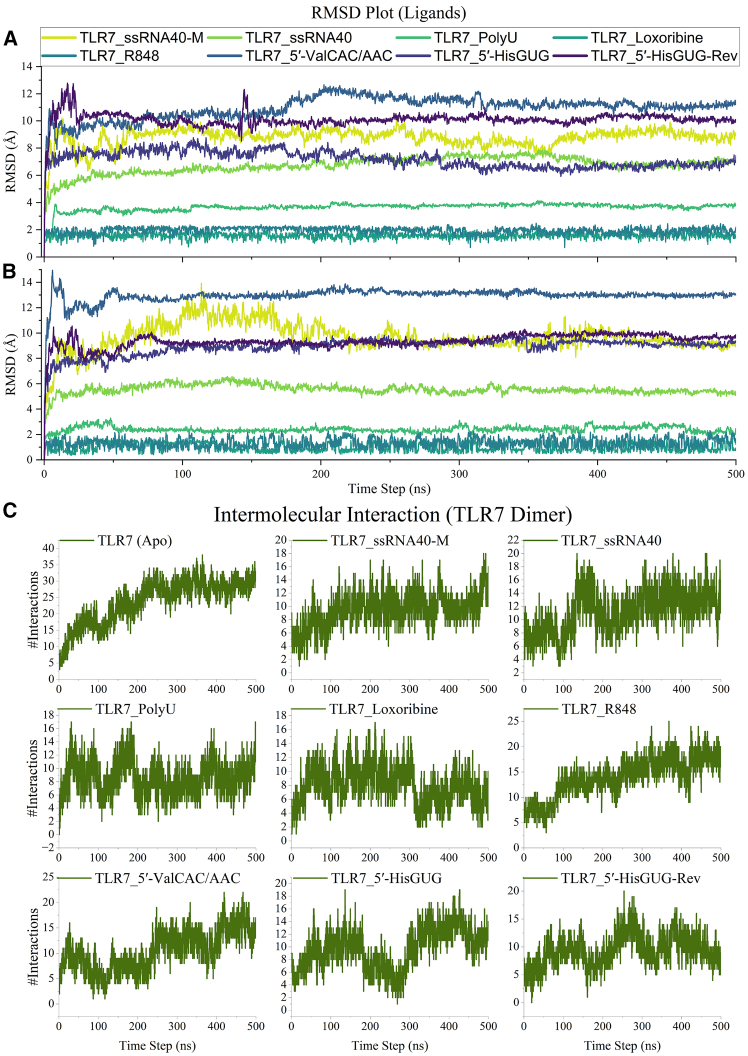
RMSD plot of ligands bound to TLR7 and MD simulations showing average number of intermolecular interactions in TLR7 dimer complexes during 500 ns. (*A*) RMSD plot of chain A and chain B. (*B*) RMSD plot for ligands during 500 ns of MD simulations shows comparable stability of various ligands, including small molecules (PolyU, loxoribine, R848) and RNAs (ssRNA40-M, ssRNA40, 5′-ValCAC/AAC, 5′-HisGUG, 5′-HisGUG-Rev). (*C*) The plot compares the interaction counts for the apo form of TLR7 and its complexes with various small molecules and RNAs. Notably, the apo form shows the highest interaction count, while R848 exhibits the highest interaction count among the ligands tested, suggesting its potential role in stabilizing the TLR7 dimer.

To gain a deeper understanding of the binding stability of various ligands to TLR7, we calculated the RMSD values for the ligands bound to chain A and chain B of TLR7 separately over 500 ns of MD simulations. The analysis aimed to compare the stability of small molecules and RNAs within the TLR7 binding pockets and to identify which ligands maintain stable binding conformations. The RMSD graph for ligands bound to chain A of TLR7 are shown in Fig. 6 *A*. The average RMSD with their standard deviation for Ligands in chain A and chain B shown in Table S4 and Table S5, respectively. The analysis revealed that PolyU RNA exhibited the lowest average RMSD of 1.5 Å with a standard deviation of 0.1 Å. This indicates that PolyU RNA maintains a highly stable binding conformation in the TLR7 binding pocket. Similarly, loxoribine also demonstrated relatively low RMSD values, with an average of 1.9 Å and a standard deviation of 0.2 Å, suggesting good stability. In contrast, R848 showed the highest average RMSD of 10.9 Å and a standard deviation of 0.8 Å, indicating significant instability and large fluctuations in its binding conformation. This suggests that R848 does not maintain a stable interaction with chain A of TLR7. The ssRNA40-M and ssRNA40s exhibited average RMSD values of 6.7 and 3.6 Å, respectively, with the ssRNA40 showing better stability. 5′-ValCAC/AAC displayed moderate stability with an average RMSD of 7.1 Å and a standard deviation of 0.5 Å. 5′-HisGUG and 5′-HisGUG-Rev, however, had higher average RMSD values of 10.1 and 9.3 Å, respectively, indicating significant instability with chain A of TLR7. Also, we have provided the RMSD graph for ligands bound to chain B of TLR7 in Fig. 6 *B*. Similar to chain A, PolyU RNA exhibited the lowest average RMSD of 1 Å with a standard deviation of 0.3 Å, indicating high stability. Loxoribine also showed good stability with an average RMSD of 1.3 Å and a standard deviation of 0.3 Å. R848 again showed the highest average RMSD of 12.9 Å with a standard deviation of 0.6 Å, confirming its unstable binding. The ssRNA40-M displayed an average RMSD of 5.5 Å with a standard deviation of 0.3 Å, while the ssRNA40 showed better stability with an average RMSD of 2.4 Å and a standard deviation of 0.2 Å. 5′-ValCAC/AAC exhibited moderate stability with an average RMSD of 8.9 Å and a standard deviation of 0.6 Å. 5′-HisGUG and 5′-HisGUG-Rev had high average RMSD values of 9.3 and 9.6 Å, respectively, indicating significant instability in their binding conformations.

### All tested ligands of TLR7 reduce intermolecular interactions between two monomeric units of TLR7

To identify molecules that stabilize the TLR7 dimer, we calculated the average number of intermolecular interactions between TLR7 chains during the 500 ns of MD simulations for both the apo form and complexes with various small molecules and RNAs. These interactions are crucial for understanding how different ligands influence the stability of the TLR7 dimer. The number of intermolecular interactions for the TLR7 dimer in different complexes are depicted in Fig. 6 *C*. The apo form of TLR7 exhibited the highest number of interactions, averaging 24 interactions. This high number of interactions in the absence of any ligand suggests that the TLR7 dimer has an inherent stability maintained through extensive intermolecular contacts. This inherent stability serves as a benchmark to evaluate the effects of various ligands. The complex with ssRNA40-M showed an average of nine interactions, a significant reduction compared with the apo form. This lower number of interactions indicates that ssRNA40-M does not significantly enhance the stability of the TLR7 dimer and might even interfere with the natural intermolecular contacts. Similarly, the ssRNA40 complex had an average of 11 interactions. This suggests that ssRNA40 contributes to the stability of the TLR7 dimer to some extent, but not as effectively as the apo form. The involvement of these RNAs within the binding pockets of both TLR7 chains and their interactions across the dimer axis may affect the natural dimerization process and the number of intermolecular interactions. PolyU RNA exhibited an average of nine interactions. Despite its high binding stability indicated by low RMSD values, PolyU RNA does not significantly increase the number of intermolecular interactions within the TLR7 dimer. This suggests that, while PolyU RNA binds stably, it does not contribute additional stabilizing contacts between the TLR7 chains and may interfere with existing ones. The loxoribine complex showed an average of eight interactions, indicating minimal effect on the stabilization of the TLR7 dimer. In contrast, the R848 complex had an average of 14 interactions, the highest among the ligands tested. This suggests that R848 significantly enhances the intermolecular interactions within the TLR7 dimer, potentially stabilizing the dimer structure. 5′-ValCAC/AAC exhibited an average of 10 interactions, indicating some stabilization of the TLR7 dimer, although not as pronounced as with R848. Similarly, 5′-HisGUG had an average of 10 interactions, suggesting a similar effect on TLR7 dimer stability as 5′-ValCAC/AAC. 5′-HisGUG-Rev showed an average of 9 interactions, indicating minimal contribution to the stability of the TLR7 dimer.

### Synthetic small molecules exhibit distinct intermolecular interactions with the TLR7 structure compared with RNA molecules

**Figure 7:**
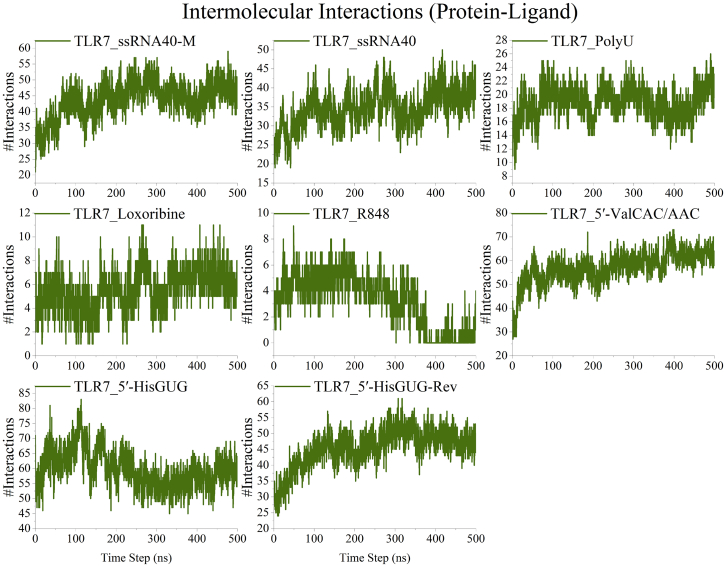
Average number of intermolecular interactions between TLR7 and various ligands (PolyU, loxoribine, R848) and RNAs (ssRNA40-M, ssRNA40, 5′-ValCAC/AAC, 5′-HisGUG, 5′-HisGUG-Rev) during 500 ns MD simulations. 5′-ValCAC/AAC and 5′-HisGUG show higher interaction counts, indicating strong potential to stabilize TLR7, while other ligands exhibit varying degrees of stabilization.

To assess the potential stabilizing effects of ligands on TLR7, we analyzed the average number of intermolecular interactions between TLR7 and various ligands (both small molecules and RNAs) during 500 ns MD simulations. These interactions provide insights into how different ligands influence the stability and interactions within the TLR7 structure. The analysis revealed distinct patterns of interaction between TLR7 and different ligands (Fig. 7). The ssRNA40-M and ssRNA40 exhibited average interaction counts of approximately 44 and 35 interactions, respectively, with TLR7. These RNA ligands showed moderate stabilization effects on TLR7, suggesting their potential role in influencing the stability and functional dynamics of the receptor. PolyU RNA, on the other hand, displayed an average of 19 interactions with TLR7, indicating a comparatively lower stabilization effect than ssRNA40-M and ssRNA40s. This suggests differential interactions of PolyU RNA with TLR7, potentially impacting its functional behavior. Loxoribine and R848 showed minimal interaction with TLR7, with average interaction counts of approximately 6 and 3, respectively. These results suggest that these small molecules may have limited direct interactions with TLR7, indicating their potential role as nonbinders or weak binders in the context of stabilizing TLR7. 5′-ValCAC/AAC and 5′-HisGUG demonstrated higher interaction counts with TLR7, averaging approximately 57 and 60 interactions, respectively. These 5′-tRNA fragments exhibited strong potential to stabilize TLR7, as indicated by their robust interaction profiles. This suggests that 5′-ValCAC/AAC and 5′-HisGUG could be effective candidates for further exploration as stabilizing ligands for TLR7. 5′-HisGUG-Rev showed a moderate stabilization effect, with an average of 45 interactions with TLR7. While slightly lower than 5′-ValCAC/AAC and 5′-HisGUG, this interaction profile still suggests a significant stabilizing potential of 5′-HisGUG-Rev on TLR7.

### Binding free energy analysis

**Figure 8:**
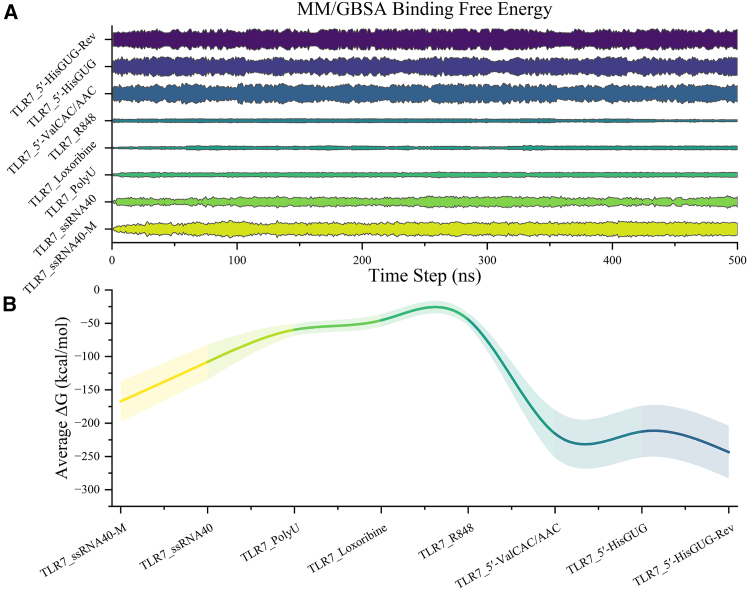
MM/GBSA binding free energy analysis of TLR7-ligand complexes. (*A*) Fluctuations in MM/GBSA binding free energy over a 500 ns molecular dynamics simulation for various TLR7-ligand complexes. Each trajectory represents the dynamic binding energy profile, highlighting the stability and variations in binding affinity over time. (*B*) The mean MM/GBSA binding free energy (ΔG) values with standard deviations for each TLR7-ligand complex. The shaded regions indicate standard deviations, demonstrating the extent of fluctuation in binding energy for each system. The results show that endogenous RNA ligands (5′-HisGUG-Rev, 5′-HisGUG, and 5′-ValCAC/AAC) exhibit the strongest binding affinities, whereas small-molecule ligands (loxoribine, R848, and PolyU) display relatively weaker interactions with TLR7.

To evaluate the stability and binding affinities of different ligands interacting with TLR7, we performed MM/GBSA binding free energy calculations over a 500 ns MD simulation. The results are presented in Fig. 8, which consists of two parts: 1) a Kite diagram (*top panel*) illustrating the fluctuations in binding free energy over time for each complex and 2) an illustrative representation of the average binding free energy (ΔG) with standard deviations (*bottom panel*), providing a comparative analysis of the ligand-TLR7 interactions. The analysis revealed substantial differences in binding free energies among the studied ligands. The highest binding affinity was observed for the 5′-HisGUG-Rev complex (−243.4 ± 39 kcal/mol), followed by 5′-ValCAC/AAC (−215.7 ± 36 kcal/mol) and 5′-HisGUG (−212.5 ± 38 kcal/mol). These results indicate that endogenous RNA ligands exhibit significantly stronger interactions with TLR7, possibly due to enhanced hydrogen bonding and stacking interactions.

In contrast, other ssRNA ligands such as ssRNA40-M (−167.3 ± 30 kcal/mol) and ssRNA40 (−108 ± 26 kcal/mol) demonstrated moderate binding affinities, with the modified ssRNA40-M showing a notable increase in stability compared with unmodified ssRNA40. Among the small-molecule ligands, PolyU (−59.7 ± 8 kcal/mol), loxoribine (−45.3 ± 9 kcal/mol), and R848 (−44.3 ± 9 kcal/mol) exhibited relatively weaker binding affinities, indicating that these molecules have a lower energetic favorability for TLR7 engagement compared with RNA-based ligands. The Kite diagram (Fig. 8 *A*) highlights the dynamic nature of binding free energy fluctuations over time. Endogenous RNA ligands, particularly 5′-HisGUG-Rev, 5′-HisGUG, and 5′-ValCAC/AAC, displayed higher fluctuations, as indicated by their larger standard deviations. This suggests that, while these ligands achieve strong binding, their interactions are more variable, likely due to structural flexibility and reorientation within the TLR7 binding site. Equally, small-molecule ligands (R848, loxoribine) and PolyU showed minimal fluctuations, reflecting relatively stable binding interactions. Similarly, the ssRNA40-M and ssRNA40 complexes displayed intermediate fluctuations, indicating that, while these ligands maintain steady interactions, modifications (as seen in ssRNA40-M) can influence the overall stability.

The MM/GBSA results underscore the superior binding affinities of endogenous RNA ligands compared with other ssRNA sequences and small-molecule agonists. The observed binding free energy trends align with the expected molecular interactions, highlighting the importance of ligand sequence and structural modifications in optimizing TLR7 targeting.

### Reverse RNA sequence of 5′-HisGUG activates endosomal TLR

**Figure 9:**
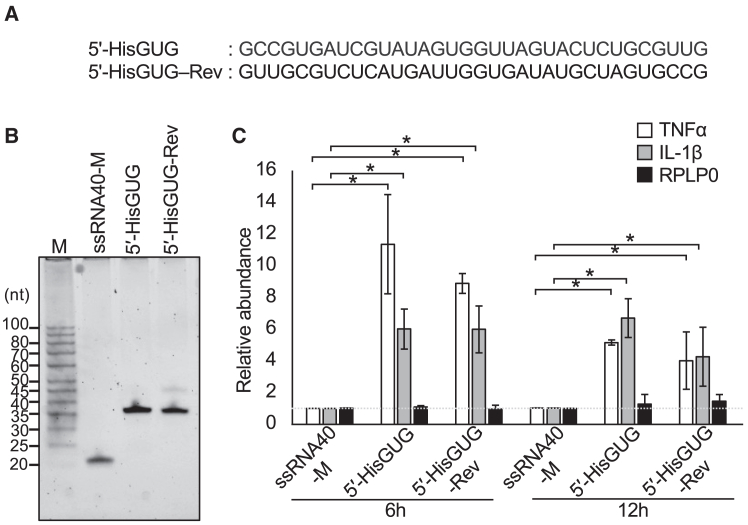
Reverse sequence of 5′-HisGUG is also an activator of TLR7. (*A*) Synthetic RNA sequences. (*B*) Indicated synthetic RNAs were synthesized by in vitro transcription, gel-purified, and analyzed with denaturing PAGE. (*C*) Using DOTAP, ssRNA40-M, ssRNA40, 5′-HisGUG, and 5′-HisGUG-Rev were transfected into HMDMs. Cells were harvested at the indicated times for RNA isolation. Total RNAs isolated from the cells were subjected to RT-qPCR for the indicated mRNAs. The quantified mRNA levels were normalized to the levels of GAPDH mRNA. For all graphs in the present study, error bars indicate mean ± SD of triplicate measurements (^∗^*p* < 0.05, ^∗∗^*p* < 0.01, and ^∗∗∗^*p* < 0.001; two-tailed *t*-test).

The 5′-HisGUG and 5′-HisGUG-Rev (Fig. 9 *A*) were synthesized in the lab by in vitro RNA synthesis. After the gel extraction, a single band of RNA (Fig. 9 *B*) was produced showing its successful production of desired RNA. To deliver the in vitro synthesized 5′-fragment of tRNA into the endosomes of HMDMs, the cells were transfected with the RNAs using the cationic liposome DOTAP. DOTAP, serving as an EV mimic, actively facilitates the delivery of encapsulated RNAs into the endosomes of recipient cells and has been widely adopted for the endosomal delivery of RNAs in previous studies (19,59,62,63). As a negative control, an inactive mutant of 20-nucleotide HIV-1-derived ssRNA, termed ssRNA40-M (9,19), was also transfected. As shown in Fig. 9 *C*, transfections of the 5′-HisGUG, as well as the 5′-HisGUG-Rev, enhanced the levels of TNF-α and IL-1β mRNAs, whereas transfections of the ssRNA40-M did not. Collectively, these results identified the 5′-HisGUG-Rev in addition to the 5′-HisGUG, as an endosomal TLR stimulating molecule.

## Discussion

Although the crystal structure of TLR7 and its RNA-binding pockets have been elucidated (10,63), the precise molecular interactions between TLR7 and its ligands remain incompletely defined. TLR7 is known to bind not only its natural RNA ligands but also non-RNA molecules, including synthetic small molecules such as loxoribine and R848 (29,49,64). It is important to note that different RNA or synthetic small-molecule ligands elicit varying activation responses, as observed previously (20). To better understand how different RNA ligands engage with TLR7 and influence its conformational dynamics, we employed a computational approach to analyze the structural fluctuations and binding free energy of these interactions. In earlier research, we discovered that two tRNA-derived 5′-fragments (5′-HisGUG and 5′-ValCAC/AAC) could activate TLR7 (19,20). In this study, we conducted MD simulations of 5′-HisGUG, 5′-ValCAC/AAC, and a reverse sequence of 5′-HisGUG (5′-HisGUG-Rev) to identify the signature interaction patterns between TLR7 and these RNA fragments. We compared these interaction results with those obtained from the interaction of TLR7 with synthetic small molecules. The binding affinity of ligands to TLR7 and the stability of ligand-TLR7 interactions over simulation time were assessed using MM/GBSA binding free energy analysis. Furthermore, our experimental results confirmed that 5′-HisGUG-Rev activates endosomal TLR7 when delivered to the endosome of HMDMs.

To accurately simulate the interactions between TLR7 and its ligands, we first refined the TLR7 model for our study. To create and establish the accurate TLR7 model, we selected the cryo-EM structure of the TLR7 dimer (PDB: 7CYN (47)) because it includes the transmembrane region. Upon analysis, we noted that the structure was missing a loop region. After modeling the missing loop, we observed that it plays a crucial role in stabilizing the dimer through intermolecular interactions. It provided valuable insights into potential ligand and RNA binding sites, while also highlighting the loop’s role in dimerization, which was critical for the functional stability of TLR7. Understanding and accurately modeling this loop region was essential for our study, as it represents an important area for drug design. Furthermore, we transferred the binding site residues from monkey TLR7 to our model TLR7. This has provided a detailed map of critical interaction points. Previously, TLR7 was modeled using the monkey x-ray complex of TLR7-R (5GMH) by removing the ligand (65). Our refined TLR7 mode with the transformed binding site not only opened avenues for exploring the dynamic interactions between TLR7 and various ligands but also it laid the groundwork for further experimental and computational studies. This can lead to the development of novel therapeutic strategies targeting TLR7-related pathways in various diseases, including autoimmune disorders and infections.

The characterization of TLR7 interactions with tRNA-derived 5′-fragments and synthetic small molecules began with molecular docking studies. We hypothesize that high binding affinity, as suggested by docking scores and an extensive interaction network, may correlate with the stabilization and activation of TLR7. However, despite the strong binding of ssRNA40-M observed in simulations, it is known to lack TLR7 activation potential. This indicates that stronger binding affinity alone is not responsible for activating TLR7; rather, the specific RNA sequence that binds to the TLR7 RNA-binding pocket plays a crucial role in determining activation potential. In contrast, ssRNA40 and PolyU RNA also demonstrated robust binding, with key interactions stabilizing the TLR7 complex. Unlike ssRNA40-M, ssRNA40 and PolyU RNA are known for their ability to activate TLR7, suggesting that its strong binding could contribute to more effective receptor activation. Conversely, the poor docking scores and limited interactions observed for 5′-HisGUG and 5′-HisGUG-Rev initially suggested that they might be ineffective in engaging TLR7. However, earlier studies showed that 5′-HisGUG activates TLR7 more strongly than ssRNA40 (19), implying that the specific sequence of 5′-HisGUG is more potent for TLR7 activation, even if its docking scores do not reflect this. Despite the low docking scores, we observed extensive intermolecular interactions between 5′-HisGUG, 5′-HisGUG-Rev, and TLR7, highlighting their potential for effective binding and functional relevance. This suggests that extensive intermolecular interactions may carry more weight than the docking score in the activation process. Moreover, there is room for optimization of 5′-HisGUG to improve its docking performance and enhance its immune response activation. Additionally, 5′-ValCAC/AAC showed preferential binding to chain B of the TLR7 dimer, suggesting specific interactions that may facilitate targeted binding, making it a promising candidate for selective RNA-based therapeutic design. While studying the docking of synthetic small molecules, we identified distinct interaction mechanisms specific to each molecule. Loxoribine, with its moderate binding affinity and involvement in π-cation interactions, exhibits a different binding mode compared with PolyU RNA. Notably, Lys470 plays a key role in mediating these interactions, suggesting that modifications at this site could either enhance or reduce loxoribine binding, offering a valuable target for drug design. In contrast, R848 showed relatively lower binding affinity and fewer interactions, indicating that it may function as a weaker TLR7 agonist. The π-cation interactions with Lys212 in chain B suggest that enhancing these interactions through structural modifications could improve R848’s binding affinity and efficacy as a TLR7 modulator. Overall, we learned that the number and type of interactions—including hydrogen bonds, salt bridges, and π-π stacking—contribute significantly to the stability and specificity of TLR7-RNA complexes, influencing their activation potential. The consistent interactions observed for Asn479, Lys212, and Asp469 across both chains underscore their importance in maintaining the integrity and stability of the TLR7-ligand complex. These residues could be critical targets for designing new ligands or modulators of TLR7 activity.

The RMSD analysis provides important insights into the dynamic behavior of TLR7 when complexed with different ligands and RNAs. While previous studies have conducted MD simulations with shorter durations (e.g., 50 ns) (65), which may not fully capture the complex’s behavior, our analysis extended the simulation to 500 ns, offering a more comprehensive time frame to examine the dynamics. This extended duration, combined with a refined TLR7 model, has resulted in more robust findings. The lower RMSD values for ssRNA40 and PolyU RNA indicate stable interactions, supporting their role in effectively stabilizing TLR7. On the other hand, the higher RMSD value for loxoribine suggests greater structural flexibility, potentially linked to weaker or less-stable binding interactions. Despite having higher docking scores, 5′-HisGUG and 5′-HisGUG-Rev demonstrate relatively low RMSD values, indicating significant stability, particularly for 5′-HisGUG, which shows stability comparable with ssRNA40. These findings suggest that the stability of the TLR7-RNA complexes is influenced by the number and nature of intermolecular interactions, as indicated by the docking results, rather than solely by RMSD values.

The RMSD analysis of ligands with individual chains (chain A and chain B) of TLR7 complexes provides crucial insights into the stability and binding affinity of different ligands. PolyU RNA consistently exhibited the lowest RMSD values across both chain A and chain B, indicating that it maintains the most stable binding conformation among all tested ligands. This strong and stable interaction suggests that PolyU RNA has a high binding affinity for TLR7, making it a potent ligand. The ssRNA40-M and ssRNA40 showed varying degrees of stability, with the ssRNA40 demonstrating better overall stability. This indicates that the ssRNA40 has a more stable interaction with TLR7 compared with the ssRNA40-M. 5′-HisGUG showed lower RMSD values for both chains compared with ssRNA40-M. This highlights the importance of considering both docking scores and RMSD values in evaluating the stability and binding affinity of ligand-TLR7 complexes. Loxoribine demonstrated relatively stable binding with low RMSD values in both chains, although with slightly higher fluctuations than PolyU RNA. This indicates a good but slightly less-stable interaction compared with PolyU RNA. R848, on the other hand, showed the highest RMSD values in both chains, indicating significant instability and suggesting weak binding affinity. This implies that R848 is not a suitable ligand for stable binding to TLR7.

To evaluate the effect of the ligand on the intermolecular interactions between the two chains of TLR7, we analyzed the number of interactions occurring between them. The high number of interactions in the apo form underscores the inherent stability of the TLR7 dimer, which is maintained through extensive intermolecular contacts. The presence of RNAs within the binding pockets of both TLR7 chains and their interactions across the dimer axis appear to reduce the natural intermolecular interactions seen in the apo form. This indicates that these RNAs may interfere with the native dimerization interface, affecting overall stability. Despite its high binding stability indicated by low RMSD values, PolyU RNA does not significantly enhance the stability of the TLR7 dimer in terms of intermolecular interactions. This suggests that, while PolyU RNA binds stably, it does not contribute additional stabilizing contacts between the TLR7 chains and may interfere with existing ones. Among all tested ligands, R848 shows the highest number of interactions, indicating its potential role in significantly stabilizing the TLR7 dimer. This could be due to its ability to induce additional intermolecular contacts within the dimer, enhancing stability. The ssRNA40 and 5′-ValCAC/AAC and 5′-HisGUG show moderate interaction counts, suggesting some degree of stabilization. However, these interactions are not as extensive as those observed in the apo form or the R848 complex. The presence of these RNAs within the binding pockets and across the dimer interface likely influences the natural dimerization and reduces the number of native interactions. These findings suggest that, while the TLR7 dimer exhibits inherent stability in its apo form, certain ligands such as R848 can further enhance this stability by increasing the number of intermolecular interactions. The presence of RNAs, on the other hand, appears to influence the dimerization interface, affecting the natural stability of the TLR7 dimer.

To further investigate the relationship between ligand binding and TLR7 activation, we conducted MM/GBSA binding free energy calculations. These analyses revealed significant differences in binding affinities, with endogenous RNA ligands (5′-HisGUG-Rev, 5′-ValCAC/AAC, and 5′-HisGUG) showing the highest affinities, likely due to enhanced hydrogen bonding and stacking interactions. In contrast, small-molecule ligands (R848, loxoribine, PolyU) exhibited notably lower binding affinities, suggesting weaker interactions with TLR7. Interestingly, although these RNA ligands demonstrated stronger interactions, they also showed higher fluctuations in free energy. In contrast, small molecules exhibited more stable binding with lower fluctuations, suggesting relatively rigid interactions with TLR7 that still lead to receptor activation. These findings indicate that, while strong binding is crucial, it does not necessarily correlate directly with activation potential. Additionally, the results emphasize that docking scores alone may not reliably predict activation, as they fail to account for the dynamic nature of ligand-TLR7 interactions. However, the combined use of MM/GBSA analysis and docking scores provides a more holistic view of ligand binding behavior, supporting the idea that both binding affinity and conformational flexibility play key roles in TLR7 activation.

Computational analysis suggests that 5′-HisGUG-Rev is a promising candidate for experimental testing of TLR7 activation. As expected, 5′-HisGUG-Rev successfully activated TLR7 when delivered into the endosomes of HMDMs. This is the first demonstration that the reverse sequence of a known TLR7-activating RNA also retains the ability to activate TLR7. However, this phenomenon needs to be validated for other RNA sequences as well. Our study offers a refined TLR7 model for computational simulations, providing a reliable method to evaluate these RNA candidates. After comparing and analyzing all the values from docking and MD simulation (Table 5), it is obvious that that the synthetic molecules (loxoribine and R848) show weak binding affinity, high RMSD, and fewer interactions, they may not be ideal candidates for further development due to potential toxicity. Among all naturally occurring and tested 5′-fragments of tRNAs, 5′-HisGUG-Rev stands out as the best candidate among the three. It has the best dimer stability (lower RMSD), the highest number of intermolecular interactions, good flexibility (lowest RMSF), and TLR7 activation potential validated through experimental studies. While 5′-VAlCAC/AAC shows strong binding to chain B and good interaction counts, it is less stable in terms of RMSD, making 5′-HisGUG-Rev the more balanced option overall for drug development and therapeutic applications.

**Table 5:** Comparative results from computational analysis

	ssRNA40-M	ssRNA40	5′-VAlCAC/AAC	5′-HisGUG	5′-HisGUG-Rev	PolyU	Loxoribine	R848	Apo (TLR7 alone)
Docking scores chain A	−253.18 kcal/mol	−163.17 kcal/mol	−44.87 kcal/mol	68.27 kcal/mol	636.33 kcal/mol	−8.414 kcal/mol	−5.843 kcal/mol	−3.908 kcal/mol	NA
Docking scores chain B	−138.76 kcal/mol	−78.58 kcal/mol	−208.69 kcal/mol	1767.50 kcal/mol	206.02 kcal/mol	−7.563 kcal/mol	−4.796 kcal/mol	3.995 kcal/mol	NA
RMSD dimer	8.029 ± 0.746 Å	6.277 ± 0.975 Å	8.962 ± 1.401 Å	6.719 ± 0.975 Å	7.681 ± 0.639 Å	6.277 ± 0.975 Å	13.356 ± 1.724 Å	8.932 ± 1.418 Å	10.330 ± 1.205 Å
RMSF	3.410 ± 1.365 Å	3.091 ± 1.642 Å	3.524 ± 1.430 Å	3.137 ± 1.247 Å	3.475 ± 1.689 Å	3.161 ± 1.588 Å	4.249 ± 2.106 Å	4.198 ± 1.765 Å	3.096 ± 1.482 Å
RMSD chain A	6.736 ± 0.661 Å	3.655 ± 0.643	7.189 ± 0.587 Å	10.104 ± 0.5296 Å	9.365 ± 0.572 Å	1.583 ± 0.188 Å	1.941 ± 0.236 Å	10.936 ± 0.864 Å	NA
RMSD chain B	5.555 ± 0.392 Å	2.401 ± 0.225 Å	8.918 ± 0.631 Å	9.365 ± 0.572 Å	9.684 ± 1.140 Å	1.033 ± 0.353 Å	1.325 ± 0.372 Å	12.952 ± 0.607 Å	NA
Intermolecular interactions (dimer)	9	11	10	10	9	9	8	14	24
Intermolecular interactions (TLR7-ligand)	44	35	57	60	45	19	6	3	NA

## Acknowledgments

We thank the SNIoE Magus02 HPC cluster facility for running the experiment. This work was supported by the 10.13039/501100001407DBT/Wellcome Trust India Alliance Fellowship (grant no. IA/E/20/1/505672) awarded to Dr. Kamlesh Ganesh Pawar. This study was also supported by SNIoE postdoctoral fellowship awarded to K.B.L. The authors are thankful to the THSTI for providing Schrodinger software for computing binding free energy calculation.

## Author contributions

K.P. conceptualized the study. K.P., A.S., and R.V. designed the study. K.B.L. and S.A. performed computational simulations. K.P. and S.J performed experiments. K.B.L., A.S., R.V., S.A., and K.P. analyzed data. K.B.L., K.P., S.A., and A.S. wrote the paper.

## Declaration of interests

The authors declare no competing interests.

## Declaration of generative AI and AI-assisted technologies in the writing process

During the preparation of this work the authors used ChatGPT, an AI language model developed by OpenAI, for assistance in editing and refining the manuscript text. After using this tool/service, the authors reviewed and edited the content as needed and take full responsibility for the content of the publication.

## Supporting material

Document S1. Tables S1–S5

Video abstract

Document S2. Article plus supporting material
