# Supplementary material for: 5′-tRNAHisGUG fragment: A preferred endogenous TLR7 ligand with reverse sequence activation insights
Source: Biophys J. 2025 May 5;124(12):1961–78. doi: 10.1016/j.bpj.2025.04.027 (PMC7617684; doi:10.1016/j.bpj.2025.04.027)
Supplement: Document S1. Tables S1–S5 [file mmc1.pdf]

Biophysical Journal, Volume *124*

Supplemental information

5′-tRNA^{HisGUG} fragment: A preferred endogenous TLR7 ligand with reverse sequence activation insights

Kiran Bharat Lokhande, Ashutosh Singh, Rajan Vyas, Shreya Joe, Shailendra Asthana, and Kamlesh Pawar

Supplementary Data:

5′-tRNA^{HisGUG} fragment: A preferred endogenous TLR7 Ligand with reverse sequence activation insights.

Running title: 5′-tRNA^{HisGUG}: Preferred TLR7 Ligand

Authors: Kiran Bharat Lokhande[1,3†], Ashutosh Singh[1†], Rajan Vyas[1], Shreya Joe[1], Shailendra Asthana[3] and Kamlesh Pawar[1,2*]

Table S1: Docking Score TLR7-Ligand Complexes.

Complex	Docking Score (kcal/mol)	
	**TLR7-Chain A**	**TLR7-Chain B**
7CYN-ssRNA40-M	-253.1	-138.7
7CYN-ssRNA40	-163.1	-78.5
7CYN-PolyU	-8.4	-7.5
7CYN-Loxoribine	-5.8	-4.7
7CYN-R848	-3.9	-3.9
7CYN-5′-ValCAC/AAC	-44.87	-208.69
7CYN-5′-HisGUG	68.27	176.50
7CYN-5′-HisGUG-Rev	636.33	206.02

Table S2: RMSD Values of C-α Atoms for TLR7 Complexes.

TLR7 (Apo)	TLR7-ssRNA40-M	TLR7-ssRNA40	TLR7_PolyU	TLR7_Loxoribine	TLR7_R848	TLR7-5′-ValCAC/AAC	TLR7-5′-HisGUG	TLR7-5′-HisGUG-Rev
10.330	8.029	6.277	6.277	13.356	8.932	8.962	6.719	7.680
±1.205	±0.746	±0.975	±0.975	±1.724	±1.418	±1.401	±0.975	±0.638

Table S3: RMSF Values of C-α Atoms for TLR7 Complexes.

TLR7 (Apo)	TLR7-ssRNA40-M	TLR7-ssRNA40	TLR7_PolyU	TLR7_Loxoribine	TLR7_R848	TLR7-5′-ValCAC/AAC	TLR7-5′-HisGUG	TLR7-5′-HisGUG-Rev
3.096	3.410	3.091	3.161	4.249	4.198	3.524	3.137	3.475
±1.482	±1.365	±1.642	±1.588	±2.106	±1.765	±1.430	±1.247	±1.688

Table S4: RMSD values for ligands bound to TLR7 Chain A during 500 ns of MD simulations.

TLR7-ssRNA40-M	TLR7-ssRNA40	TLR7_PolyU	TLR7_Loxoribine	TLR7_R848	TLR7-5′-ValCAC/AAC	TLR7-5′-HisGUG	TLR7-5′-HisGUG-Rev
8.786	6.736	3.655	1.583	1.941	10.936	7.189	10.103
±0.661	±0.643	±0.320	±0.188	±0.236	±0.864	±0.587	±0.5296

Table S5: RMSD values for ligands bound to TLR7 Chain B during 500 ns of MD simulations.

TLR7-ssRNA40-M	TLR7-ssRNA40	TLR7_PolyU	TLR7_Loxoribine	TLR7_R848	TLR7-5′-ValCAC/AAC	TLR7-5′-HisGUG	TLR7-5′-HisGUG-Rev
9.684	5.555	2.401	1.033	1.325	12.952	8.918	9.365
±1.140	±0.392	±0.225	±0.353	±0.372	±0.607	±0.631	±0.572
